# Differential effects of domesticated and wild *Capsicum frutescens* L. on microbial community assembly and metabolic functions in rhizosphere soil

**DOI:** 10.3389/fmicb.2024.1383526

**Published:** 2024-07-01

**Authors:** Can Wang, Yinghua Zhang, Shaoxiang Wang, Xia Lv, Junqiang Xu, Xueting Zhang, Qing Yang, Fanlai Meng, Bin Xu

**Affiliations:** ^1^College of Landscape and Horticulture, Yunnan Agricultural University, Kunming, Yunnan, China; ^2^Institute of Medicinal Biological Technique, Wenshan Academy of Agricultural Sciences, Wenshan, Yunnan, China; ^3^College of Biological and Agricultural Sciences, Honghe University, Mengzi, Yunnan, China

**Keywords:** *Capsicum frutescens* L., rhizosphere soil, metagenome, metabolome, *Massilia*

## Abstract

**Objective:**

Rhizosphere microorganisms play crucial roles in the growth and development of plants, disease resistance, and environmental adaptability. As the only wild pepper variety resource in China, domesticated *Capsicum frutescens* Linn. (Xiaomila) exhibits varying beneficial traits and affects rhizosphere microbial composition compared with its wild counterparts. In this study, we aimed to identify specific rhizosphere microbiome and metabolism patterns established during the domestication process.

**Methods:**

The rhizosphere microbial diversity and composition of domesticated and wild *C. frutescens* were detected and analyzed by metagenomics. Non-targeted metabolomics were used to explore the differences of metabolites in rhizosphere soil between wild and domesticated *C. frutescens*.

**Results:**

We found that the rhizosphere microbial diversity of domesticated variety was significantly different from that of the wild variety, with *Massilia* being its dominant bacteria. However, the abundance of certain beneficial microbes such as *Gemmatimonas, Streptomyces, Rambibacter,* and *Lysobacter* decreased significantly. The main metabolites identified in the wild variety included serylthreonine, deoxyloganic acid, vitamin C, among others. In contrast, those identified in the domesticated group were 4-hydroxy-l-glutamic acid and benzoic acid. Furthermore, the differentially enriched pathways were concentrated in tyrosine and tryptophan biosynthesis, histidine and purine-derived alkaloids biosynthesis, benzoic acid family, two-component system, etc.

**Conclusion:**

This study revealed that *C. frutescens* established specific rhizosphere microbiota and metabolites during domestication, which has important significance for the efficient utilization of beneficial microorganisms in breeding and cultivation practices.

## 1 Introduction

Humans have changed the genotypes and characteristics of crops through the process of domestication, resulting in desired traits such as larger fruits and higher active ingredient content. However, domestication also leads to a decrease in plant genetic diversity, compromising the inherent capacity of crops to resist diseases and insect pests (Raaijmakers and Kiers, 2022; Ran et al., 2023). As integral components of the rhizosphere biosystem, rhizosphere microorganisms play vital roles in nutrient cycling, plant health, and soil ecology (Sahu et al., 2017; Garcia and Kao-Kniffin, 2018). As an interactive and interdependent organic complex, soil rhizosphere microorganisms and plants jointly determine the growth, development and yield of crops. Plant species are important factors affecting the structure and function of rhizosphere microbial communities (Nakayasu et al., 2022). The roots of distinct plant varieties selectively shape unique bacterial communities, and the repeated cultivation of plants from the same variety in a particular field can further enhance the specific enrichment of rhizosphere bacteria by these plants (Smalla et al., 2001). Host plant types, tissue genotypes and crop domestication exert great influence on plant-associated microorganisms (İnceoğlu et al., 2010). Among them, crop domestication is a biological evolution process regulated by human activities, which can significantly impact the structure, diversity and function of the rhizosphere microbial community (Liu et al., 2020; Yue et al., 2023). The transition of plants from their native habitats to cultivated soils has also led to great changes in the composition of rhizosphere microflora (Pérez-Jaramillo et al., 2018). Domestication of plants plays a pivotal role in shaping both the assembly and metabolic functions of the rhizosphere microbiome (Abdullaeva et al., 2022; Gutierrez and Grillo, 2022; Yue et al., 2023). A study on the rhizosphere bacterial and fungal communities of domesticated and wild tetraploid wheat has showed that the rhizosphere microbial community is influenced by host genes and domestication status. Domesticated and wild wheat varieties have formed different rhizosphere microbial diversity, composition and functions (Yue et al., 2023). Plant domestication has a profound impact on the composition and metabolic function of rhizosphere microbiota. Previous studies have shown that corn domestication and genetic improvement may increase the diversity of corn rhizobium and significantly change the composition of rhizobium community (Huang et al., 2022). These changes enhance plant adaptability to biological stress and improve the supply of nutrients to plants. In addition, other studies have suggested that wild/native species possess a strong ability to adapt to harsh environments, whereas domestication may have a negative impact on relevant beneficial traits in the rhizosphere microbial community (Pérez-Jaramillo et al., 2016; Spor et al., 2020). This may be due to the deliberate selection of specific protective mechanisms during the process of domestication, resulting in plants that are unable to effectively recruit relevant beneficial microorganisms. However, it is not clear what microbes and functions are lost during the domestication of most plants. The relative abundance of *Glomeraceae, Streptomyces, Bradyrhizobium,* and *Rhizobium* in the rhizosphere community of wild rice was much higher than that of cultivated rice (Shi et al., 2018). This suggests that wild crops had better resistance to pathogenic bacteria than cultivated ones. To sum up, a comprehensive exploration of the structure and function of rhizosphere microorganisms in local habitats and their interspecific domestication is conducive to a deeper understanding of the interaction between plants and rhizosphere microorganisms during domestication. Understanding how plant domestication establishes specific rhizosphere microbiome and metabolic functions is of great significance for the efficient utilization of beneficial microorganisms in future agricultural development.

Capsicum Linn. is an annual or short-lived perennial herb of the Solanaceae family. After domestication and cultivation, it is used as a special spice and seasoning, spreading to North America, Africa, Europe, and Asia (Kang et al., 2001). Capsicum contains a variety of vitamins essential for human health, including a notably high content of vitamin C, therefore earning its name “the king of vitamin C.” It also contains protein, flavonoids, phenols, carotene, peroxidase and cellulose and other substances, all of which play important roles in delaying human aging, improving human resistance and enhancing body endurance (Batiha et al., 2020). Because of its strong adaptability, Capsicum has gradually become one of the most important vegetables widely cultivated in the world, with China being the largest producer (Zhang et al., 2023). *Capsicum frutescens* Linn. (Xiaomila) is the only wild pepper variety resource in China, with abundant germplasm resources in both quantity and species (Wang et al., 2023). However, due to the long-term artificial selection, as well as the cultivation and popularization of new varieties of *C. frutescens*, the relevant beneficial traits in the rhizosphere microbial community have shown different effects. At present, more attention is diverted to plant traits and functions that can effectively regulate beneficial microorganisms in the rhizosphere during the selection of new crop varieties. For instance, the strategic selection of plants that effectively enrich beneficial microbial communities while inhibiting pathogenic groups could potentially reduce the necessity of incorporating disease resistance into plant genomes (Mendes et al., 2013; Olanrewaju et al., 2019).

Therefore, exploring beneficial rhizosphere microorganisms may have positive impacts on crop breeding and agricultural production, highlighting their importance as a key area for future research and development.

This study used metagenomics and metabolomics to explore the regulatory mechanisms of wild and artificial cultivated *C. frutescens* on the structure and function of rhizosphere microbial community. By elucidating the interaction between *C. frutescens* growth and rhizosphere microorganisms during domestication, we aimed to identify specific rhizosphere microbiome and metabolism patterns established during this process. This research offers a novel approach with significant implications for the efficient utilization of beneficial microorganisms in future Capsicum breeding and cultivation practices.

## 2 Materials and methods

### 2.1 Study site

**Figure 1:**
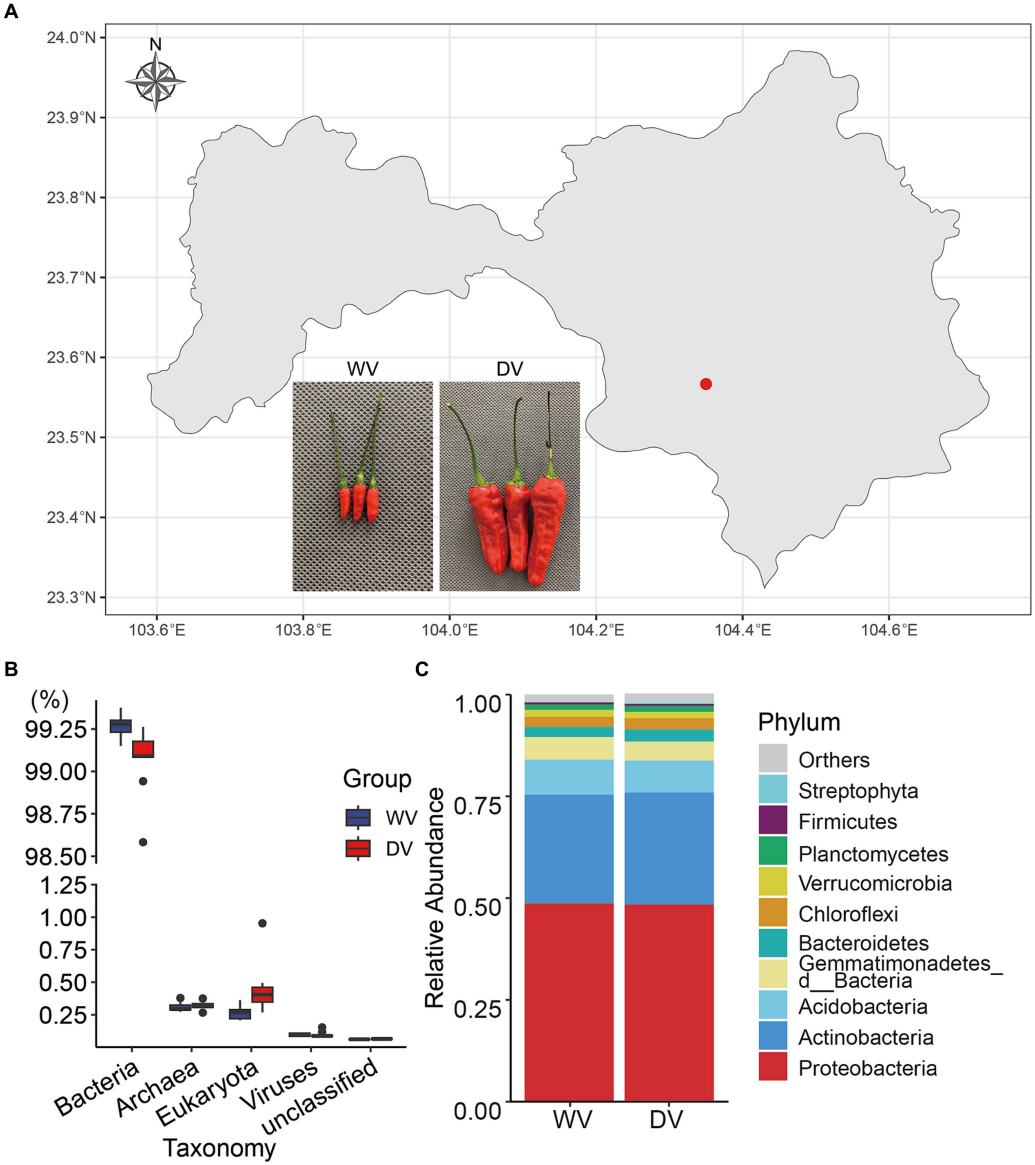
Map of the soil sampling site and overall composition of rhizosphere microorganisms in the wild variety (WV) and domesticated variety (DV) *C. frutescens*. **(A)** Map of the soil sampling site in Yanshan City, Yunnan Province, China. **(B)** Relative abundance of soil microorganisms at the kingdom level. **(C)** Relative abundance of soil microorganisms of the top 10 phyla.

The experimental site in this study was located in the Plateau Characteristic Vegetable Planting Base (longitude 104°21′19″ E, latitude 23°34′47” N) in Wenshan City, Yunnan Province. Positioned at a low latitude (altitude 1528.2 m), the site fell within the south subtropical region. The wild variety (WV) and the domesticated variety (DV) of *C. frutescens* were planted in close proximity within the same field. Soil types and cultivation management measures were maintained consistently for both varieties (Figure 1A). Rhizosphere soil samples were collected during the picking season in September 2022, following the method described by Tan et al. (2017). Plant roots and other impurities were removed. The soils were stored at −80°C until experiment. Each sample had nine replicates.

### 2.2 Soil DNA extraction and sequencing

The Mag-Bind^®^ Soil DNA Kit (Omega Bio-tek, United States) was utilized to extract high-quality total genomic DNA from a 500 mg soil sample, ensuring optimal extraction efficiency. DNA integrity was detected by 1% agarose gel electrophoresis. DNA was segmented by Covaris M220 (Gene Co. Ltd., China), and then fragments of about 400 bp were selected. The libraries were constructed using the NEXTFLEX Rapid DNA-Seq (Bioo Scientific, United States). Paired-end sequencing with short reads (2*150 bp) was performed using Illumina NovaSeq 6,000 platform (Illumina, San Diego, CA, United States).

### 2.3 Metagenomic analysis

The raw data were filtered using the fastp (v.0.20.0) software (Chen et al., 2018). Metagenome assembly was conducted using the software MEGAHIT (v.1.1.2). Contigs smaller than 300 bp were removed. The open reading frame (ORFs) was predicted and filtered using the MetaGene (v. 2.6.3) software, removing those smaller than 100 bp (Hyatt et al., 2010). Non-redundant (NR) gene sets were constructed based on the longest ORF using the CD-HIT (v. 4.6.1) software (Fu et al., 2012). Gene abundance data were obtained using SOAPaligner (v.2.21) (Li et al., 2008). The amino acid sequences of non-redundant gene sets were aligned with various databases for taxonomy and gene function annotation using Diamond software (v.0.8.35) (Buchfink et al., 2015). Species annotations were obtained from the NR database, followed by the calculation of species abundance using the sum of the corresponding gene abundance for each species. Gene functions were annotated using the Kyoto Encyclopedia of Genes and Genomes database (KEGG: https://www.genome.jp/kegg) and the Comprehensive Antibiotic Resistance Database (CARD).

The vegan (v. 2.4.0) and ggplot2 (v. 3.4.4) packages were used for alpha diversity and statistical analysis. The Chao richness index estimates the total number of species in a community by considering rare and abundant species (Chao and Shen, 2003). The ACE index (Abundance-based Coverage Estimator) also assesses species richness but adjusts for undersampling (Chao and Lee, 1992). The Shannon index measures both species richness and evenness in a community, with higher values indicating greater diversity (Shannon, 1948). The Simpson diversity index quantifies the probability that two individuals randomly selected from a sample belong to different species, with lower values suggesting higher diversity (Simpson, 1949). These indices were used to describe the alpha diversity features of rhizosphere microbial communities. Wilcoxon rank-sum test was used to compare the composition of rhizosphere microorganisms between the two soils. The non-parametric factorial Kruskal–Wallis (KW) sum-rank test was used to identify noteworthy distinctions in taxonomic compositions at the phylum or genus levels between the two groups of soil samples. We applied linear discriminant analysis (LDA) to assess the magnitude of the influence of species abundance on differential effect. Principal coordinates analysis (PCoA) was conducted using Bray–Curtis distance as a metric to compare beta diversity. The co-expression network was constructed using the Gephi (v. 0.10.1) software based on Pearson correlation.

### 2.4 Metabolite extraction and liquid chromatography tandem-mass spectrometry (LC–MS/MS) analysis

Briefly, 50 mg of soil sample was placed into a 2 mL centrifuge tube along with a 6 mm diameter grinding bead. Metabolites were extracted using 400 μL of extraction solution (methanol: water = 4:1, v:v) containing 0.02 mg/mL of internal standard (L-2-chlorophenylalanine). The samples were then ground using a Wonbio-96c frozen tissue grinder (Shanghai Wanbo Biotechnology Co., Ltd.) for 6 min at −10°C and 50 Hz, followed by low-temperature ultrasonic extraction for 30 min at 5°C and 40 kHz. After incubating the samples at −20°C for 30 min, they were centrifuged for 15 min at 4°C and 13,000 *g*. The supernatant was transferred to an injection vial for LC–MS/MS analysis.

The LC–MS/MS analysis of the sample was performed using a Thermo UHPLC-Q Exactive HF-X system equipped with an ACQUITY HSS T3 column (100 mm × 2.1 mm i.d., 1.8 μm; Thermo Fisher Scientific, United States). The mobile phases comprised 0.1% formic acid in water:acetonitrile (95,5, v/v) (solvent A) and 0.1% formic acid in acetonitrile:isopropanol:water (47.5,47.5, v/v) (solvent B). The flow rate was set at 0.40 mL/min, and the column temperature was maintained at 40°C.

The mass spectrometric data were acquired utilizing a Thermo UHPLC-Q Exactive HF-X Mass Spectrometer (Thermo Fisher Scientific, United States) featuring an electrospray ionization (ESI) source operating in both positive and negative modes. Experimental parameters were optimized as follows: source temperature (425°C); sheath gas flow rate (50 arb); auxiliary gas flow rate (13 arb); ion-spray voltage floating (ISVF) was configured at -3500 V for negative mode and 3,500 V for positive mode; normalized collision energy ranged from 20 to 60 V with a rolling scheme for MS/MS at 20–40-60 V; full MS resolution was set at 60000, while MS/MS resolution was set at 7500.

### 2.5 Metabolomics data analysis

The MassHunter Workstation (version v10.0.707.0) software was used to analyze the raw data generated by LC–MS/MS. Data were filtered to remove noise, column bleed, and derivatized reagent peaks, and further matched with public databases such as NIST (version 2017), Fiehn (version 2013), and MS-DIAL (version 2021). Statistical analyses were performed using the R software (R version R-4.3.1). Principal component analysis (PCA) and orthogonal least partial squares discriminant analysis (OPLS-DA) were performed using R package “ropls” (Version 1.6.2). Significantly differential metabolites (DIMs) were identified by considering both the variable importance in the projection (VIP) obtained from the OPLS-DA model and the *p*-value derived from Student’s *t*-test. DIMs were identified with VIP value >1, *p* < 0.05, and fold-change (FC) ≥ 2 or FC ≤ 0.5. KEGG pathway enrichment analysis was used to identify significant pathways associated with these DIMs. A pathway was considered enriched when the ratio *x*/*n* > *y*/*N*, indicating a substantial representation of DIMs within the pathway, and when the *p* < 0.05, signifying statistical significance in the enrichment.

## 3 Results

### 3.1 An overview of the metagenomics and microbial communities

In this study, high-throughput sequencing was used to identify the rhizosphere soil microorganisms of different varieties of *C. frutescens* (Figure 1A). A total of 267.6388 Gb clean data were obtained. The assembled 16.782 Gb bases belonged to 25,557,714 contigs, and the average length of contigs was 678 bp. The results of taxonomy annotation indicated that the majority of the genes belonged to bacteria (Figure 1B). To better understand structural differences between the two microbial communities, we further analyzed overall relative abundances at the phylum level. The results showed that the combined relative abundances of Proteobacteria and Actinobacteria accounted for more than 75% of the bacteria presented in both the DV and WV rhizosphere soils, indicating their dominance in both environments (Figure 1C). However, no significant differences were observed in the bacterial community structure at the phylum level between the DV and WV rhizosphere soils. The fungal phyla with the highest relative abundance were Ascomycota, Mucoromycota and Basidiomycota, collectively constituting over more than 90% of all fungal communities (Supplementary Figure S1A). At the genus level (Supplementary Figure S2), we found that in the DV group, *Sphingomonas* took up 2.81%, *Pseudolabrys* 2.52%, *Streptomyces* 2.04%, and *Ramlibacter* 1.79%, while in the WV group, *Sphingomonas* took up 2.98%, *Pseudolabrys* 2.57%, Streptomyces 2.23%, and *Ramlibacter* 2.40%. Through Wilcoxon signed-rank test of gene abundance, we found that the relative abundances of *Ramlibacter*, *Gemmatirosa*, *Occallatibacter*, *Mesorhizobium,* and *Lysobacter* in the WV soil were significantly higher than those in the DV soil (*p* < 0.01), while those of *Massilia* and *Ferruginibacter* were significantly lower than those of WV (*p* < 0.05). *Rambibacter, Gemmatimonas*, and *Lysobacter* are beneficial microorganisms that promote soil and plant health, and the decrease of their abundances in the DV soil indicated that artificial cultivation may favor specific groups of microbes while suppressing others. At the genus level, the relative abundances of *Aspergillus*, *Penicillium*, *Rhizophagus*, *Monosporascus* in fungi were notably higher in both groups. However, these molds and arbuscular mycorrhizal (AM) fungi were significantly lower in the DV rhizosphere soil compared with those of WV.

### 3.2 Alpha diversity of the microbial community in the rhizosphere soil

**Figure 2:**
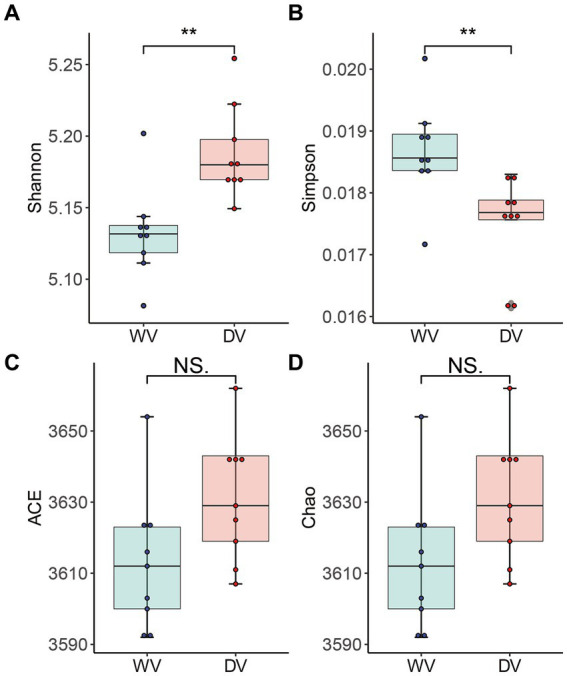
Alpha diversity of rhizosphere microorganisms in WV and DV *C. frutescens*. Shannon **(A)**, Simpson **(B)**, ACE **(C)**, and Chao **(D)** indices in the WV and DV. The level of significance between WV and DV is indicated as ** (*p* < 0.01) based on Student’s *t*-test.

The α diversity of rhizosphere soil microbial communities of the WV and DV *C. frutescens* was evaluated by richness index (Chao and ACE index) and diversity index (Shannon and Simpson index). The Shannon index in the DV group was significantly higher than that of WV (*p* < 0.01) (Figure 2A), while the Simpson index of DV was significantly lower than that of WV (*p* < 0.01) (Figure 2B). We observed no significant difference in the Chao and ACE indexes between the two groups (Figures 2C,D). This indicated that domestication significantly altered the diversity of rhizosphere microorganisms, although microbial richness remained affected.

### 3.3 Variations in beta diversity caused by domestication

**Figure 3:**
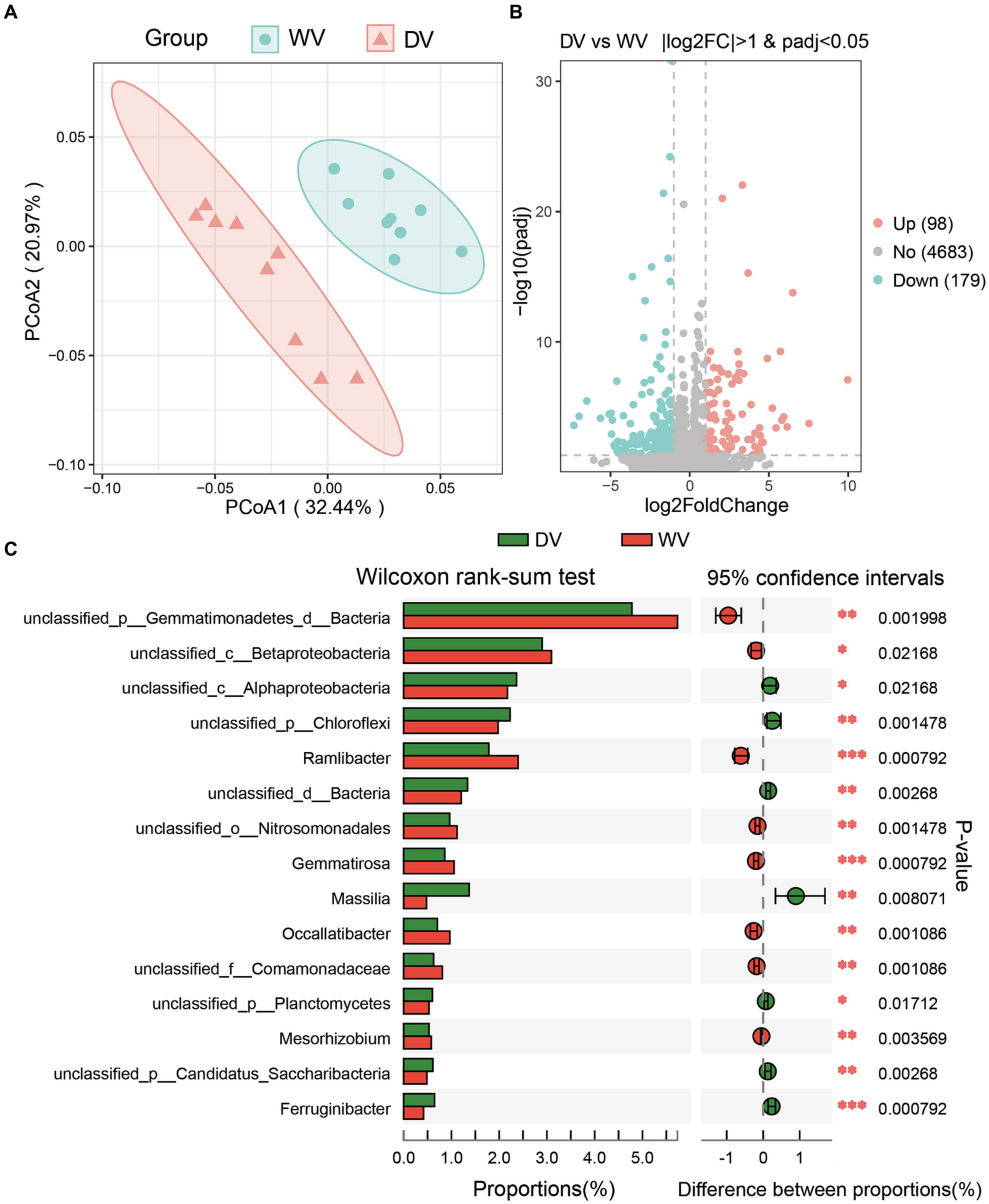
Differences in rhizosphere soil microorganisms caused by domestication. **(A)** Principal co-ordinates analysis (PCoA) showed the differences in soil microorganisms of WV and DV. **(B)** Differential analysis showing abundance disparities between DV and WV *C. frutescens*. **(C)** 15 differential taxa between DV and WV *C. frutescens* identified by Wilcoxon rank-sum test.

The PCoA was conducted to assess the overall differences in microbial community structure of the two soils. The results showed that microbial communities from all samples could be well separated into two groups, with PCoA1 explaining 32.44% of the total variance, and PCo2 20.97% (Figure 3A). This may suggest a potential significant disparity in the rhizosphere soil microbial community between the DV and WV *C. frutescens*. A further differential analysis revealed that the relative abundances of 98 genera were significantly upregulated, while 179 were significantly downregulated (Figure 3B). Domestication significantly decreased the relative abundances of rhizosphere soil microbes. By Wilcoxon rank sum test, 15 differential taxa were identified between the WV and DV soils, such as *Gemmatimonadetes*, Betaproteobacteria, Alphaproteobacteria, etc. (Figure 3C). These findings indicated that the artificial cultivation of *C. frutescens* may change the microbial composition of its rhizosphere soil.

### 3.4 Biomarker rhizosphere microorganisms and functional gene annotation of wild and domesticated *C. frutescens*

**Figure 4:**
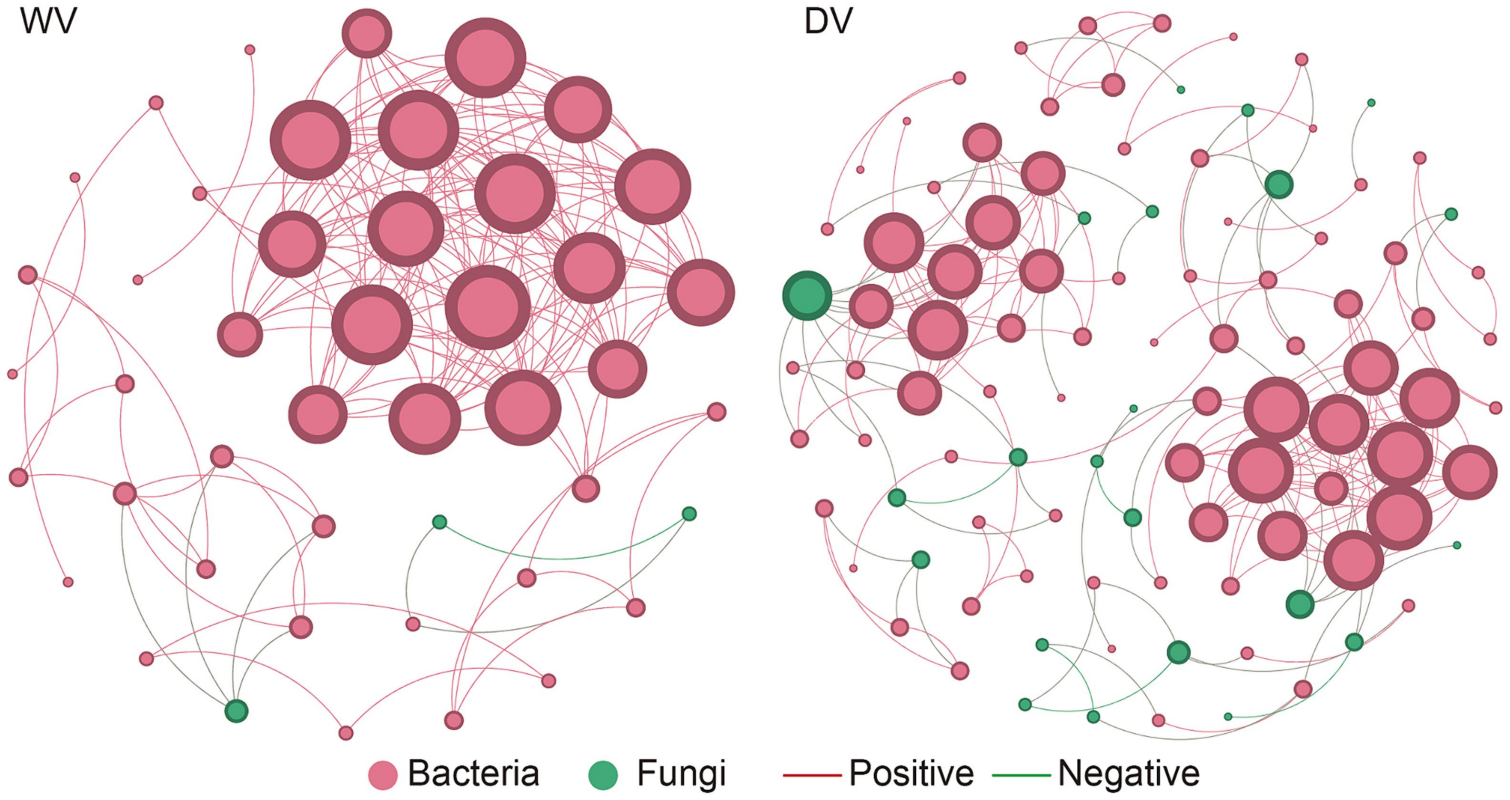
Co-occurrence network of genes in DV and WV *C. frutescens*. Red circles represent bacteria and green circles represent fungi. Red lines represent positive correlation and green lines represent negative correlation.

LEFSE analysis was then conducted to identify microbial biomarkers indicative of differences between rhizosphere microorganisms in the soils (Supplementary Figure S3). We found that *Massilia*, *Nocardia*, *Ferruginibacter*, *Rhizomicrobium*, *Caulobacter*, and *Sphaerobacter* were among the most abundant bacterial genera in the DV group. In contrast, *Ramlibacter*, *Occallatibacter*, *Lysobacter*, *Gemmatirosa*, *Azospirillum*, *Gemmatimonas,* and *Panacibacter* were detected to be significantly enriched in WV group. In summary, these microbial biomarkers may respond to cultivation patterns to varying degrees, leading to the differences between soil samples. An analysis of the microbial symbiotic network revealed noticeable evolution in the interaction patterns of microorganisms during the domestication process of *C. frutescens*. Additionally, the core microorganisms varied between the wild and domesticated microbial networks. Specifically, the DV microbial network showed higher connectivity between bacteria and fungi. In contrast, the WV microbial network had higher bacteria-bacteria connectivity. In addition, the network connectivity between bacteria and bacteria, bacteria and fungi in the DV microbial network was higher than that of the WV network (Figure 4). The DV network highlighted an obvious increase in negative correlations among microorganisms than the WV network. Furthermore, the dominant groups in each network confirmed the effects of *C. frutescens* domestication on microbial interactions. These results showed that domestication led to an increase in the correlation between bacteria and fungi, but decreased the correlation between bacteria in the rhizosphere soil of *C. frutescens.*

### 3.5 Effects of domestication on the function of rhizosphere microbial community

**Figure 5:**
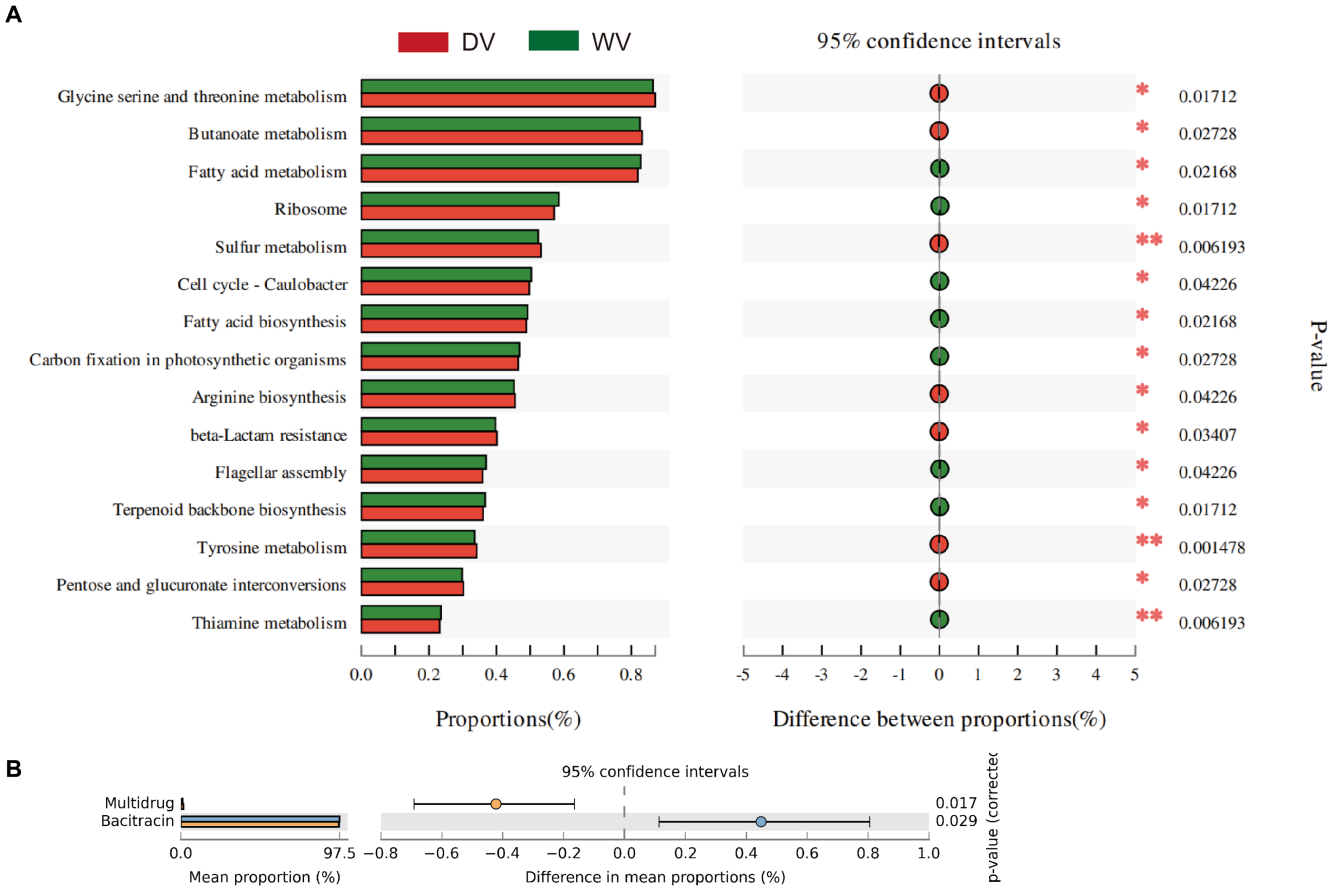
Gene functions of rhizosphere microorganisms in DV and WV *C. frutescens*. **(A)** Significant enriched pathways are screened based on Wilcox non-parametric test. **(B)** Differential enrichment of resistance genes between DV and WV.

The composition of microorganisms is often closely related to their functions. KEGG enrichment analysis was therefore performed on the differential genes between WV and DV groups (Figure 5A). It was found that metabolites in the DV soil had significantly higher levels of fatty acid metabolism, fatty acid biosynthesis, carbon fixation in photosynthetic organisms, and thiamine metabolism, but lower levels of glycine serine and threonine metabolism, butanoate metabolism, sulfur metabolism, arginine biosynthesis and tyrosine metabolism than those in WV soil. These results indicated that the cultivation of *C. frutescens* promoted the metabolism of fatty acids, but inhibited the metabolism of various amino acids. The reduction of amino acids may adversely impact the biosynthesis of proteins and thus affect the growth of plants to some extent. We further annotated and analyzed antibiotic resistance genes based on the CARD database. The results showed that the abundance of multidrug resistance genes in the DV soil was significantly lower than that in WV soil (*p* < 0.05), while the expression of bacitracin was higher (*p* < 0.05) (Figure 5B). Indicating that the abundance of antibiotic resistance genes was affected by domestication.

### 3.6 Metabolite analysis of the rhizosphere soil

**Figure 6:**
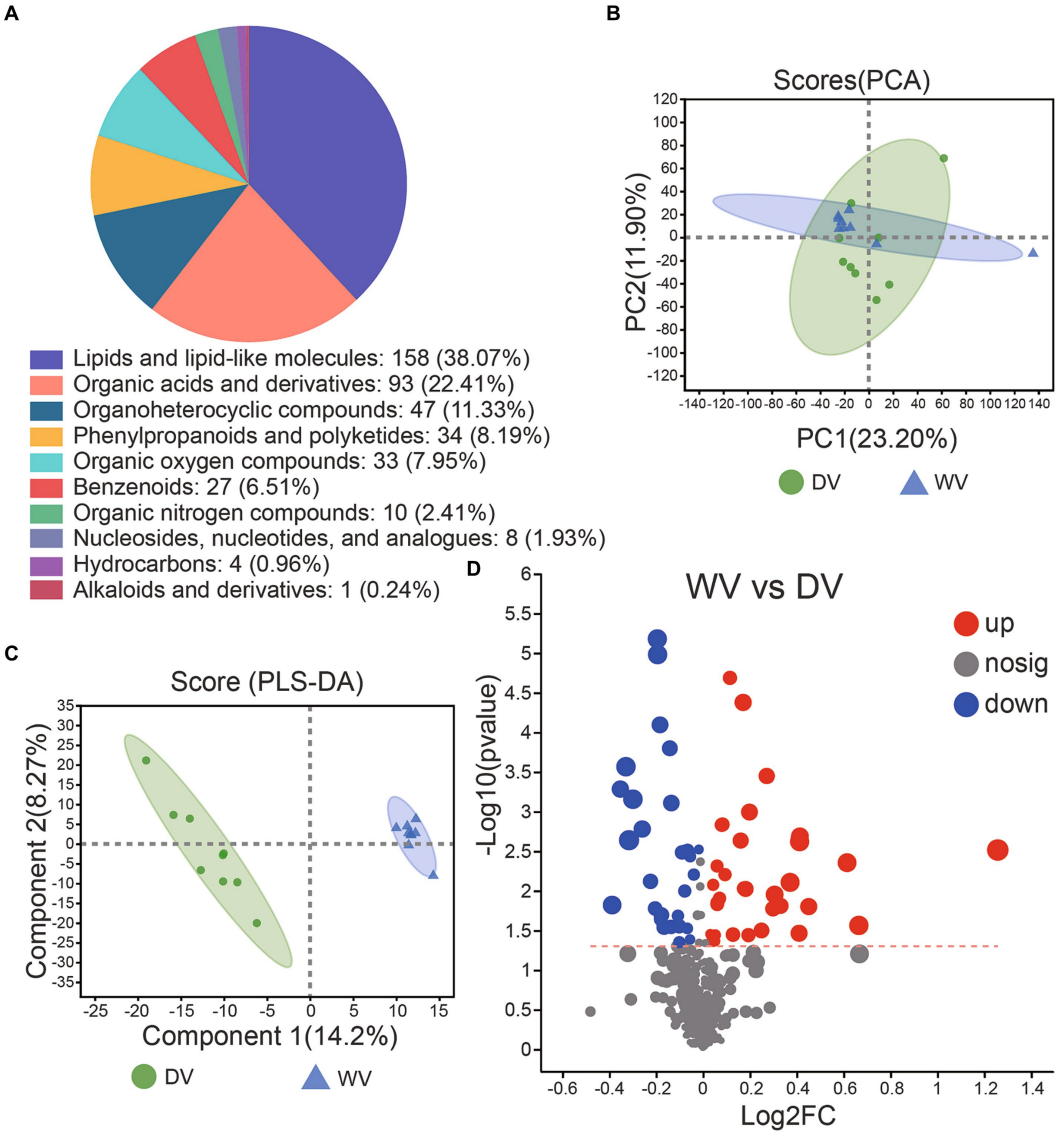
Composition and distribution of metabolites in DV and WV soils. **(A)** Overall metabolite distribution in the DV and WV groups. **(B)** PCA plots of the GC-TOFMS metabolites. **(C)** Two hundred randomly arranged substitution tests were performed between DV and WV in the OPLS-DA model. **(D)** Volcano diagram of differential metabolites between the two groups.

The soil microbiome is intricately connected to the presence and composition of metabolites. To unravel metabolic characteristics of different groups of soils, we performed non-targeted metabolomics analysis using GC-TOF-MS. A total of 17,145 metabolites were identified, including 7,896 positive ion patterns and 9,249 negative ion patterns. Subsequent overall cluster analysis on the identified metabolites revealed that the metabolite composition of the soil was mainly concentrated in lipids and lipid-like molecules, organic acids and derivatives, organoheterocyclic compounds, phenylpropanoids and polyketides, as well as benzenoids (Figure 6A). PCA was conducted to clarify the relationship between metabolite levels and soil samples. An obvious separation was observed, indicating differences in the abundances of metabolites between soils. To be specific, 22.30% of the variances were explained by PC1 while 11.50% by PC2 (Figure 6B). We then carried out OPLS-DA to get deeper insights into the discrimination and separation between metabolites of the two soils. R2Y value was 0.7551, and the corresponding Q2 value was −0.3362, indicating that the model was reliable and the data were stable and accurate. The two groups were clearly separated, while the variations explained by PC1 and PC2 were 13.7 and 13.5%, respectively (Figure 6C). Through further differential analysis, we identified 31 upregulated and 28 downregulated metabolites in the DV soil compared with the WV soil (Figure 6D).

### 3.7 Identifying DIM between WV and DV rhizosphere soils through non-targeted metabolomics

**Figure 7:**
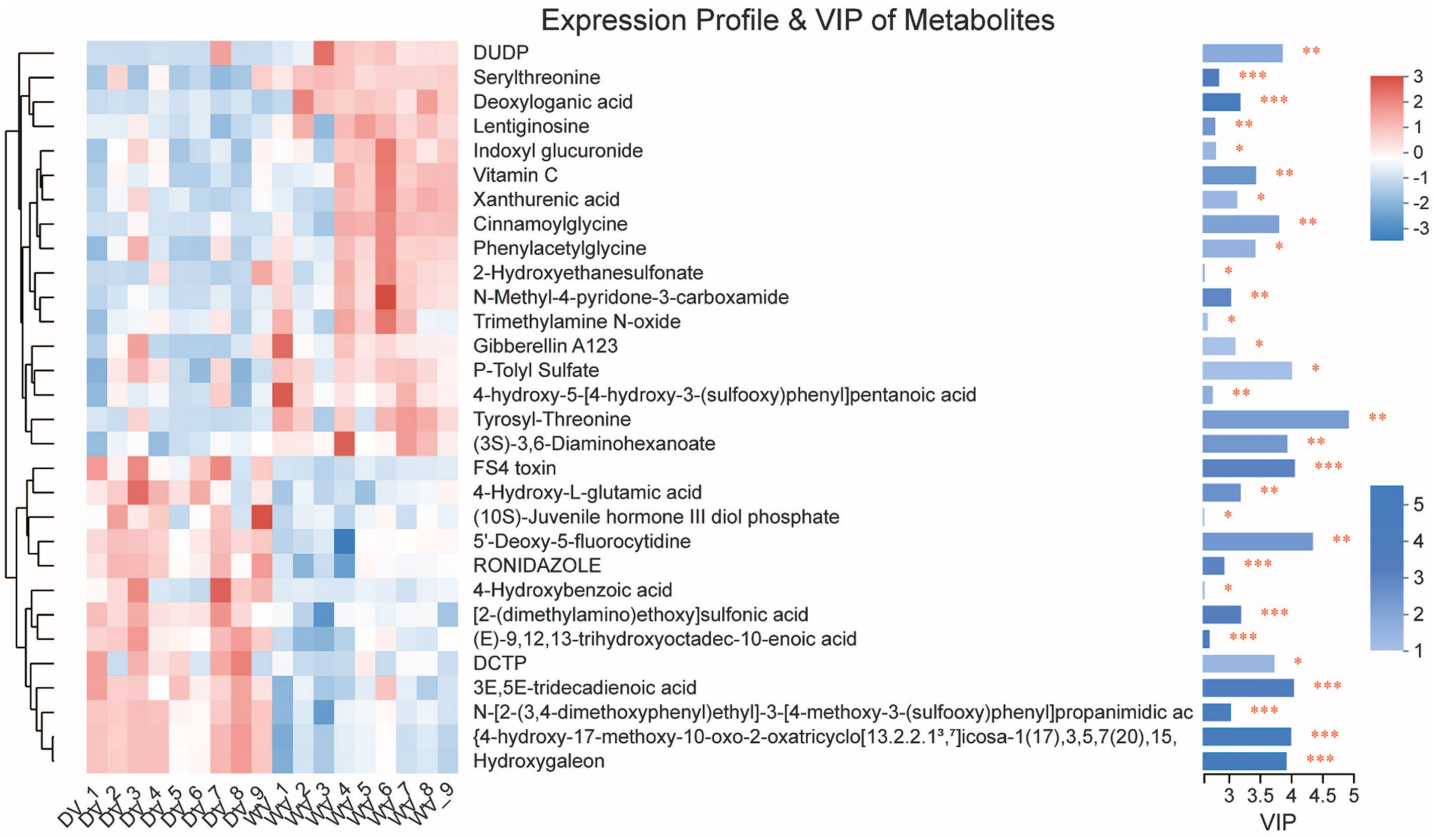
Metabolite profiling of DV and WV. The color blocks in different positions represent the relative accumulation of metabolites in corresponding positions. Red indicates a high accumulation of metabolites and blue indicates a low accumulation of metabolites.

Further statistical analysis was carried out to identify a total of 30 significant DIMs between the WV and DV soils. The main metabolites of the WV group were serylthreonine, deoxyloganic acid, lentiginosine, indoxyl glucuronide, vitamin C, xanthurenic acid, cinnamoylglycine, and phenylacetylglycine, while those in the DV group included 4-Hydroxy-l-glutamic acid, 4-Hydroxybenzoic acid, 2-(dimethylamino) ethoxy sulfonic acid and 3E,5E-tridecadienoic acid (Figure 7). Simultaneously, the downregulation of multiple amino acids may suggest a shift from anabolic to catabolic processes in the domesticated variety, potentially impairing protein biosynthesis and impacting plant growth.

## 4 Discussion

Based on metagenomic technology, the rhizosphere microbial diversity and composition of wild and domesticated varieties *C. frutescens* were detected and analyzed. It was found that the rhizosphere microbial diversity of domesticated *C. frutescens* was higher than that of wild variety, with the dominant bacteria being *Massilia* and *Ferruginibacter*. The differentially enriched rhizosphere microorganisms of the wild variety were identified as *Gemmatimonas*, *Rambibacter*, *Lysobacter*, etc. Further analysis of metabolites and functional pathways of the two varieties based on metabolome technology showed that the cultured variety exhibited higher levels of glutamate metabolism, but significantly lower levels of vitamin C and amino acids such as serylthreonine, cinnamoylglycine and phenylacetylglycine.

The functions of *Massilia* have generated attention due to its wide distribution, strong adaptability to the environment and potential value in various applications. It helps soil repair, enzyme production, and promotion of pepper growth and metabolism (Ofek et al., 2012; Ran et al., 2023; Xu et al., 2023). Feng et al. (2016) isolated *Massilia putida* (*M. putida*) 6NM-7 from ore in tungsten mining area, which has potential application value in the prevention and control of soil-borne diseases and the development of new soil fumigants. Studies have shown that by increasing the relative abundance of Massilia and Terrimonas, disease resistance and root development of chili peppers can be promoted for a healthy growth in microplastics contaminated soils. Some strains of *Massilia* also have potential application value in repairing soil contaminated by PAHs such as phenanthrene. In addition, it also has the ability to resist a variety of heavy metals (Lou et al., 2016; Wang et al., 2016; Zheng et al., 2017). Ran et al. found that *Massilia* was enriched in pepper planted in soil contaminated by microplastics. Through redundancy analysis of the bacterial community and pepper growth indicators, it was found that *Massilia* was positively correlated with plant height, root biomass and above-ground biomass of pepper at seedling stage (Ran et al., 2023). *Massilia* has antagonistic effects against Phytophthora *in vitro* and can enhance plant disease resistance (Ofek et al., 2012). It can also promote plant growth by producing and releasing plant hormones, such as auxin and gibberellin (Ganesh et al., 2024). These results indicate that *Massilia* may be closely related to the growth of spicy millet, inhibiting the growth and invasion of pathogenic bacteria, while improving the resistance of pathogenic bacteria. This is of great significance for enhancing the resistance and resistance of plants. *Ferruginibacter* belongs to denitrifying bacteria and has not been reported to be enriched in Capsicum.

In addition, compared with the DV group, the WV group was rich in active microorganisms that are beneficial to plants, such as *Gemmatimonas*, *Ramlibacter,* and *Lysobacter*, and the abundance of these microorganisms in the cultivated variety was significantly lower. *Gemmatimonas* is one of the dominant and most influential genus of bacteria in agro-ecosystems (Banerjee et al., 2016a,b), and are particularly abundant in soils with high organic matter content (Li et al., 2012). It plays a key role in the release of organic carbon through the degradation of complex cellulose and lignin molecules (Banerjee et al., 2016b), and is beneficial for many legumes such as soybeans. Guo et al. (2016) have shown that *Gemmatimonas* participate in important biological metabolism of organic carbon and play a key role in carbon sequestration. *Gemmatimonas* may have a greater capacity to utilize available carbon sources than other soil microorganisms. However, it was found in this study that the gene abundance of photosynthetic organisms for carbon sequestration in the DV soil was lower than that in the WV soil, which may be related to the combined action of *Gemmatimonas* and other rhizosphere microorganisms. In addition, Shen et al. (2019) found that *Gemmatimonas* and *Lysobacter* were negatively correlated with *F. oxysporum*, suggesting that they might be involved in disease inhibition. Although the precise disease-suppressing function of *Gemmatimonas*, which are widespread in a variety of terrestrial and aquatic habitats, remains unknown, their higher abundances are often detected in healthy mass and/or rhizosphere soils with low incidence of soil-borne diseases (Yin et al., 2013). *Ramlibacter* and *Lysobacter* are well-known plant beneficial bacteria that can effectively control multiple plant diseases and promote the growth of many plant species (Zucchi et al., 2008; Shakeel et al., 2018). Among them, *Lysobacter* has a strong application prospect in the biological control of pests and diseases. It can not only colonize in the rhizosphere of various plants, but also secrete a variety of antibiotics, extracellular hydrolase and biological surface-active substances to inhibit the growth of bacteria, so as to control plant diseases. At the same time, it has inhibitory activity against many nematodes, and plays an important role in biological control (Hayward et al., 2010). In addition, studies have found that *Lysobacter* C3 can induce host plants to develop resistance to scab caused by Fusarium, and also has a good control effect on leaf spot disease (Kobayashi et al., 2005). The study of Christensen and Cook (1978) has revealed that *Lysobacter* has unique sliding and bacteriolytic activity, properties that confer significant advantages in biological control against pathogens and nematodes. To sum up, domesticated *C. frutescens* may have weaker abilities than its wild counterpart in controlling specific pathogens or pests.

Further metabolome-based analysis found that wild variety of *C. frutescens* have higher levels of coumarin, vitamin C, serine, threonine, and glycine than domesticated variety. Coumarin compounds are a very important class of natural compounds in nature. They have anti-cancer, anti-inflammatory, anticoagulant and antibacterial activities, and are therefore widely applied in medicine (Bhattarai et al., 2021). Their agricultural biological activities are also increasingly recognized. They exhibit significant agricultural antibacterial effects, particularly against pathogens such as *Phytophthora capsici*, powdery mildew, *Botrytis cinerea*, wheat scab, and others (Stringlis et al., 2019). Coumarin compounds, as benzocaine secondary metabolites synthesized by plants, exhibit antimicrobial activity and regulate the interaction between pathogenic bacteria and plants. They also exert UV protection effects. They can rapidly accumulate at the infection sites of pathogenic fungi, bacteria and viruses, and under the irradiation of ultraviolet rays from the sun, so as to aid in plant defense against infection (Lay and Anderson, 2005; Mokdad-Bzeouich et al., 2015).

Amino acids are not only essential substrates for protein biosynthesis in cells, but also participate in metabolic pathways and regulation of carbon and nitrogen balance in plants. As signaling molecules, some amino acids display special biological significance for plant development. They also serve as nutrients to promote the growth of plant roots and other organs, which is conducive to improving plant ability of absorbing and transporting nutrients (Sharma and Dietz, 2006; Moormann et al., 2022). Studies showed that the application of threonine improved the growth of corn roots and increased the concentration of nitrogen and phosphorus in roots, suggesting that threonine could promote the absorption of various nutrients and root growth of plants (Pantigoso et al., 2023). Amino acids can not only promote plant growth, but also improve the adaptability of plants to external abiotic stresses (Yobi et al., 2019). When plants encounter external stress conditions, amino acids can enhance the adaptive response of plants to various stresses by changing physiological metabolism, or regulating the expression of related genes and the activity of key enzymes in plants. l-glutamate has been shown to promote plant growth and increase bacterial population density. Additionally, it has been found to mitigate damage caused by salt stress. These findings suggest that l-glutamate plays a crucial role in tolerance to salt stress with core microbiota (Niu et al., 2022). Therefore, sufficient amino acid supply may promote plant adaptation to various environmental stresses, therefore improving crop yield and quality. In this study, it was found that the levels of threonine and glycine in the WV soil were higher than those in the DV soil, but the level of glutamate in DV was significantly higher than that in WV. Glutamic acid, as an essential amino acid, is not only an important substrate for the synthesis of other amino acids and proteins, but also affects or regulates important molecular physiological processes in plants.

This study also noted a lower level of ascorbic acid (vitamin C) in domesticated variety *C. frutescens*. Peppers contain the highest amount of vitamin C among all types of vegetables. Vitamin C is a vital micronutrient for plant growth, playing a major role in plant-environment interactions, and defense against pathogens and oxidative stress. Additionally, it is essential for plant cell division, cell wall expansion, regulation of photosynthesis and other developmental processes (Smirnoff and Wheeler, 2000). Vitamin C is also an important antioxidant for humans and has been linked to the prevention of several diseases. Three synthesis pathways have been identified in *C. frutescens* for vitamin C production. The primary pathway is the Smirnoff-Wheeler pathway (l-galactose pathway), supplemented by two bypass pathways: the myo-inositol pathway and the glucuronate pathway. The significant decrease of vitamin C level in the domesticated variety may be closely related to alterations in the above pathways. The limitation of our study lies in its single study site, which restricted the sample size and generalizability of our findings. We believe future studies could benefit from multi-site investigations to validate and extend our observations across diverse environmental contexts. Overall, our study sheds light on the intricate microbial dynamics and biochemical profiles associated with the artificial cultivation of *C. frutescens*.

## 5 Conclusion

In summary, this study found that the artificial cultivation of *C. frutescens* specifically promoted the colonization of bacteria such as *Massilia*, which promotes the growth and root biomass of pepper, and enhances the disease resistance of plants. However, artificial cultivation also suppressed beneficial microorganisms such as *Gemmatimonas*, *Ramlibacter,* and *Lysobacter*, as well as the levels of vitamin C, threonine and glycine. Conversely, the level of glutamate was found to be higher compared with the wild variety. The results showed that domesticated *C. frutescens* had certain advantages in pepper growth and development, and tolerance to specific stress factors. However, it demonstrated lower levels of disease resistance, pest resistance and certain quality attributes compared with the wild variety.

## Data availability statement

The datasets presented in this study can be found in online repositories. The names of the repository/repositories and accession number(s) can be found at: https://www.ncbi.nlm.nih.gov/, PRJNA1065111.

## Author contributions

CW: Formal analysis, Visualization, Writing – original draft, Writing – review & editing. YZ: Investigation, Software, Writing – original draft. SW: Methodology, Project administration, Writing – review & editing. XL: Formal analysis, Methodology, Writing – review & editing. JX: Formal analysis, Visualization, Writing – review & editing. XZ: Data curation, Methodology, Writing – original draft. QY: Formal analysis, Investigation, Project administration, Writing – review & editing. FM: Formal analysis, Writing – original draft, Writing – review & editing. BX: Conceptualization, Formal analysis, Funding acquisition, Writing – review & editing.
