# Supplementary material for: Differential effects of domesticated and wild Capsicum frutescens L. on microbial community assembly and metabolic functions in rhizosphere soil
Source: Front Microbiol. 2024 Jul 1;15:1383526. doi: 10.3389/fmicb.2024.1383526 (PMC11261347; doi:10.3389/fmicb.2024.1383526)
Supplement: Supplementary file 1 [file Data_Sheet_1.docx]

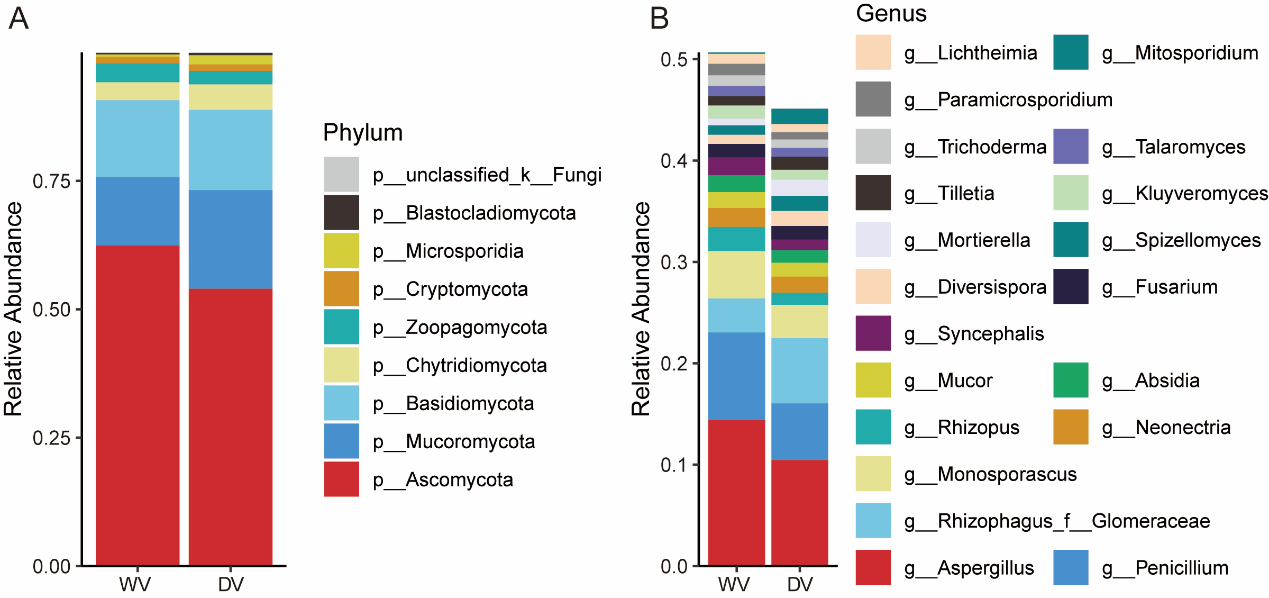


**Figure S1 Composition of rhizosphere fungi in DV and WV *Capsicum frutescens* at the phylum (A) and genus (B) level.**


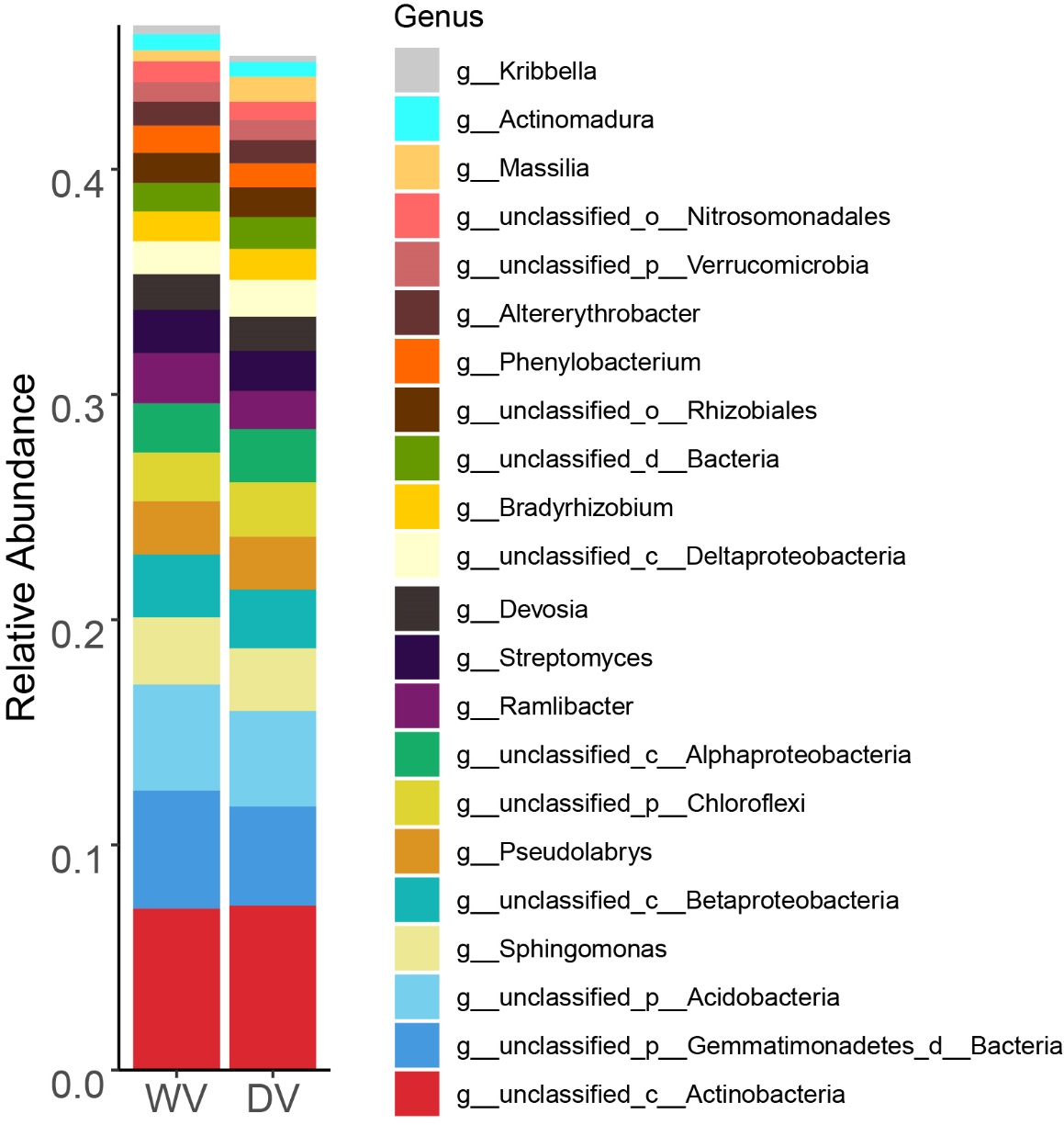


**Figure S2 Composition of rhizosphere bacteria in DV and WV *Capsicum frutescens* at the genus level.**


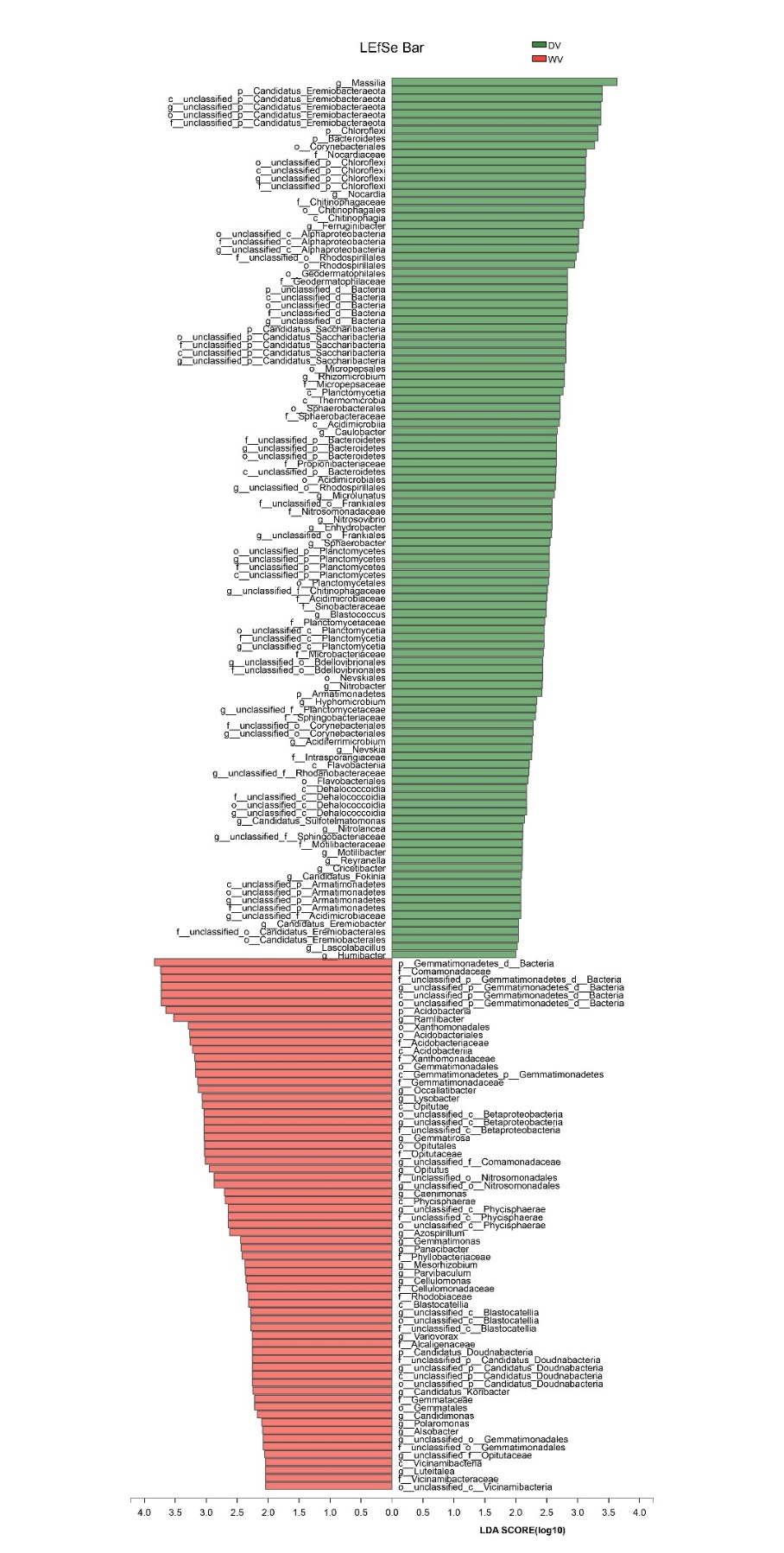


**Figure S3 Microbial biomarkers in rhizosphere microorganisms in DV and WV *C. frutescens*.**

Taxonomic cladogram obtained from LEfSe in the two groups. Biomarker taxa are highlighted by shaded areas. The cutoff value of ≥2.0 is used for LDA.
